# The central role of the Thalamus in psychosis, lessons from neurodegenerative diseases and psychedelics

**DOI:** 10.1038/s41398-023-02691-0

**Published:** 2023-12-13

**Authors:** Marco Onofrj, Mirella Russo, Stefano Delli Pizzi, Danilo De Gregorio, Antonio Inserra, Gabriella Gobbi, Stefano L. Sensi

**Affiliations:** 1 Behavioral Neurology and Molecular Neurology Units, Center for Advanced Studies and Technology - CAST, Institute for Advanced Biomedical Technology-ITAB University G. d’Annunzio of Chieti-Pescara, Chieti, Italy; 2 Department of Neuroscience, Imaging, and Clinical Sciences, University G. d’Annunzio of Chieti-Pescara, Chieti, Italy; 3 Division of Neuroscience, Vita-Salute San Raffaele University, Milan, Italy; 4 Neurobiological Psychiatry Unit, McGill University, Montreal, QC Canada

**Keywords:** Molecular neuroscience, Psychiatric disorders

## Abstract

The PD-DLB psychosis complex found in Parkinson’s disease (PD) and Dementia with Lewy Bodies (DLB) includes hallucinations, Somatic Symptom/Functional Disorders, and delusions. These disorders exhibit similar presentation patterns and progression. Mechanisms at the root of these symptoms also share similarities with processes promoting altered states of consciousness found in Rapid Eye Movement sleep, psychiatric disorders, or the intake of psychedelic compounds. We propose that these mechanisms find a crucial driver and trigger in the dysregulated activity of high-order thalamic nuclei set in motion by ThalamoCortical Dysrhythmia (TCD). TCD generates the loss of finely tuned cortico-cortical modulations promoted by the thalamus and unleashes the aberrant activity of the Default Mode Network (DMN). TCD moves in parallel with altered thalamic filtering of external and internal information. The process produces an input overload to the cortex, thereby exacerbating DMN decoupling from task-positive networks. These phenomena alter the brain metastability, creating dreamlike, dissociative, or altered states of consciousness. In support of this hypothesis, mind-altering psychedelic drugs also modulate thalamic-cortical pathways. Understanding the pathophysiological background of these conditions provides a conceptual bridge between neurology and psychiatry, thereby helping to generate a promising and converging area of investigation and therapeutic efforts.

## The missing link between psychiatry and neurology: the “Maladie expérimentale”

In 1919, after the end of World War I, a pandemic caused millions of deaths in the following five years. The pandemic was due to an H1N1 influenza A virus inaccurately named “Spanish Flu” [1, 2]. Following the acute phase of Von Economo-Cruchet Encephalitis (also known as Von Economo Disease), a significant percentage of surviving patients developed a neurological syndrome named Post-encephalitic Parkinsonism (PP) [3, 4]. Renowned neuropsychiatrists of those days (Neurology and Psychiatry were still a unified discipline) described the disease’s behavioral, emotional, and cognitive aspects in detail. PP symptoms included dementia, psychoses with delusions and complex hallucinations, disinhibition, impulsivity (including hypersexuality, kleptomania, gambling), and a variety of behavioral disorders ranging from distractible neurological symptoms (i.e., “*histrionic*” interpretations of motor disorders) to mannerisms and catatonia [5–8]. These clinical features were acknowledged as hysteria, a condition that just a few decades before was the focus and conceptual driver of the seminal psychodynamic theories put forward by Sigmund Freud [9].

The appearance of PP offered, for the first time, evidence that hysteria could be a symptom of a specific neurological condition promoted by a defined underlining pathology. PP was, therefore, named “Experimental disease/Maladie experiméntale” by the enthralled neuropsychiatrists [5, 6, 10–13]. The choice of the term “experimental” was primarily driven by the hope that the investigation of PP’s neuropathological underpinnings could shed light on the neurobiology of hysteria. Unfortunately, neuropathologists failed to provide a pathology-dependent hypothesis or mechanism for hysteria. The study of hysteria-like phenomena in PP faded away and was eventually forgotten. The topic occasionally resurfaced in authoritative neuropsychiatric handbooks until the second half of the last century [6, 14]. Ultimately, the hysteria field returned to the realm of psychodynamic theories.

In recent years, the syndromic complex of Parkinson’s Disease (PD) has moved away from the narrow field of Movement Disorders. Given the complex and multifaceted neuropsychiatric symptoms, successful attempts have been made to reclassify PD and Dementia with Lewy Body (DLB) as neuropsychiatric diseases [15, 16]. Recently, a consensus was reached to include Somatic Symptom Disorders (SSD, now termed Functional Neurological Disorders, FND) among the neuropsychiatric symptoms of PD-DLB [17–19].

Furthermore, the recent blooming of neuroimaging techniques has allowed a leap forward in understanding the functioning and anatomical substrates of the neuronal networks that modulate and affect the clinical expression of synucleinopathies [20–27].

Of note, the current COVID-19 pandemic, post-COVID, and long-COVID complications support the overwhelming incidence of neurologic and psychiatric complications associated with viral infections [28–30]. The consistency of PP has been recently challenged [8], moving from after-the-fact considerations that some cases could have been triggered by forms of genetic parkinsonism unknown or not testable at the time. The challenge can now be applied to the COVID-19 pandemic. The burden posed to health care by FND-SSD and psychiatric disorders in post-COVID patients, along with the “epidemic of Medically Unexplained Syndromes” [31, 32], underlines the need for proper understanding and modeling of the phenomena.

## Psychotic phenomenology: PD-DLB psychosis complex vs. psychedelics

The PD-DLB psychosis complex [33, 34] includes a triad of symptoms: hallucinations, mainly in the visual modality (Visual Hallucinations, VH) [34–36], delusions [33], and FND-SSD [37–40]. Many studies indicate that visual hallucinations are a clinical manifestation predictive of underlying α-synuclein deposits [41] and are considered a core criterion for diagnosing DLB [42]. VH may start as simple distortions of perceived images (“illusions”), frequent pareidolic phenomena, or extracampine hallucinations. At this stage, the symptoms can still be controlled and/or suppressed by refocusing attention. With the disease progression and the ensuing brain atrophy along with structural damage, there is an enhancement in the content complexity [33] and the building up of a more structured narrative, thereby generating complex, dreamlike, and paraphrenic (i.e., highly elaborated) presentations [43, 44], which may eventually lead to a restricted state of consciousness that lasts for days until treatment is initiated [45]. These complex manifestations prelude or are concurrent with the onset of dementia [46].

Delusions are inner, stable beliefs appearing as unmodifiable distorted thought contents unattainable by reality-checking and consensual verification. Delusions may be present in the early or advanced stages of PD and precede or are concurrent with the appearance of dementia [33, 42, 47]. Patients already exhibiting cognitive decline suffer from somatic delusions that include the perception of body deformation, the feeling of being physically controlled by external agents or being affected by allergies or invaded by parasites, or having necrotic or liquefied internal organs (the last two signs also being known as critical features of the Ekbom and Cotard delusions) [33, 38, 40].

FND-SSD are multiform symptoms belonging to the Somatic Symptom Disorder Diagnostic and Statistical Manual of Mental Disorders-5^th^ edition (DSM-5) class, typically featured by sensory, motor, or cognitive disturbances not relying on an organic basis but instead on a psychiatric one [48]. In pure SSD, patients also exhibit disproportionate concerns about their symptoms. FND-SSD prevalence in PD is 16-60% (depending on the diagnostic framing) [18, 40]. FND-SSD often precedes the appearance of PD symptoms by several years and is associated with an unfavorable prognosis. [17, 37, 38, 40, 49, 50] Over time, with the progression of the motor symptoms, FND-SSD typically subside, only to re-emerge at later stages, merging with somatic delusions [38, 40, 51]

Intriguingly, parkinsonian psychotic symptoms largely overlap with altered states of consciousness triggered by the intake of psychotropic agents like psilocybin, lysergic acid diethylamide (LSD), ketamine, and others.

LSD is a synthetic compound that induces visual, somatic, and auditory hallucinations [52]. Moreover, LSD may affect the perception of “self”. Thus, depersonalization, ego distortion, and oceanic boundlessness are frequently reported [52, 53]. This alteration, along with the impairment of the reality check monitoring system, may also provoke other dissociative symptoms akin to some FND-SSD features [51, 54]. Distortion in time perception is also reported [52, 55, 56]. Similar symptoms are reported after the intake of psilocybin [57, 58], an alkaloid tryptamine present in some mushrooms, such as *psilocybe cubensis mexicana*. LSD and psilocybin also generate rapid and profound mood and cognitive changes [59, 60].

VH occur in one-third of patients who underwent ketamine administration [61]. Intriguingly, a similar prevalence has been reported for smell and taste hallucinations, while auditory hallucinations are less frequent [61] (see Supplementary Materials). Finally, dreamy, oneiric states are commonly reported upon psychedelic intake [61–63], but similar conditions are also described in long-lasting psychotic episodes, i.e., “crepuscular states” experienced by Parkinsonian patients [36]. In advanced stages of PD or DLB, or upon precipitating factors like hospitalization, systemic conditions, or surgery, patients may enter into clouded and oneiric states of consciousness with the production of continuous or sub-continuous complex hallucinations that can lead to severely altered behavioral features that last for days, if appropriate management is not promptly ensued [34, 36].

Finally, delusions are not a common effect of psychedelics intake [64]. This apparent discrepancy is discussed in the predictive model (see Section “Towards the resolution of the mind-brain conflict”).

Given the phenomenological similarities between the PD-DLB psychosis complex and the subjective effects occurring upon psychedelic administration, in the following sections, we discuss and highlight potential similarities and shared molecular and large-scale pathophysiological pathways of neurodegeneration-driven psychosis and transitory psychotic symptoms induced by pharmacological compounds (see also Supplementary Materials – Section “The missing link between psychiatry and neurology: the “Maladie expérimentale”).

## The core model

The proposed model is supported by studies in patients suffering from pathological α-synuclein aggregates or subjects exposed to psychedelic compounds. The model is centered on the defective inhibition of structures that guarantee the top-down modulation of perception, the recruitment of priors, and the intrusion of self-centered narratives in the perceptive experience.

Studies with functional Magnetic Resonance Imaging (fMRI) have documented a critical role in the abnormal functioning of the Default Mode Network (DMN) in α-synucleinopathies and psychedelic states alike. The alterations mainly involve increased connectivity and overactivation of the Posterior Cingulate Cortex (PCC), the primary posterior hub of the DMN [22, 26, 65, 66]. Notably, the DMN activity is anti-correlated with the Task-Positive Networks (TPN) [67]. A delicate balance between these systems is needed for reality-checking and to focus attention [68]. In detail, the coupling between the DMN and TPN allows for a seamless transition between introspective self-referential thinking and externally focused attention. When we engage in self-referential thinking, the DMN generates internal representations of reality based on memories, experiences, and expectations. However, to ensure the accuracy of these representations, the TPN needs to periodically assess them against the external environment. Thus, the coupling between the DMN and TPN is involved in reality-checking by enabling the interplay between internal self-referential thinking and externally focused attention. This dynamic interaction permits to evaluate the accuracy of our perceptions, thoughts, and beliefs in relation to the external world and make appropriate adjustments when necessary. Disruptions in the balance between these networks are therefore associated with alterations in consciousness and reality perception, as in psychotic states. The thalamus is a fundamental orchestrator of the balanced activity of these networks. Therefore, the dysrhythmic thalamic pacing of the cortical activity (also known as ThalamoCortical dysrhythmia, TCD) can impair the systems’ coupling (Supplementary Materials, Section “Psychotic phenomenology: PD-DLB psychosis complex vs. psychedelics”). The DMN decoupling from the TPN may result in inappropriate activation, ultimately generating random connectivity motifs, which project internal narratives into reality monitoring and interpreting.

We propose that the TCD, characterized by improper theta rhythm (4–7 Hz) production, impairs the brain metastability as well as cortico-cortical modulations exerted by the thalamus, thereby leading to the overactivation of the DMN, being ThalamoCortical pathways also included in the DMN. TCD is also paralleled by concurrent thalamic dysfunctions that promote unbalanced filtering of external and internal information flow.

## The thalamocortical pathways and thalamocortical dysrhythmia

### Anatomy of the thalamocortical circuits

Researchers have long hypothesized that the thalamus is critically involved in the onset of altered states of consciousness, such as psychosis and hallucinations. The region can also modulate the subjective effects of psychedelics and dissociative anesthetics.

The thalamus contains ThalamoCortical relay neurons (TCRN) organized in nuclei (i.e., sensory, motor, associative) innervated by excitatory fibers arising from the infralimbic cortex (IFL). These nuclei send excitatory projections to regionally distinct columnar cortical areas (mostly layers IV and VI). Thalamic nuclei containing TCRN are also classified as first-order structures that receive sensory information (such as the lateral geniculate nucleus, receiving primary inputs from the visual system) and higher-order thalamic nuclei, which shape complex conscious states through acquiring and integrating signals from first-order Cortico-Thalamo-Cortical pre-processing, and then relaying information back to the cortex (such as the MedioDorsal nucleus of the Thalamus, MDT) [69] TCRN project excitatory axon collaterals to GABAergic neurons of the Thalamic Reticular Nucleus (TRN) (see Box 1), a structure mainly innervated by excitatory CorticoThalamic (~70%) and ThalamoCortical (~20–25%) fibers. Topographical reciprocity can be found between the cortical and TCRN neurons, and some overlap as to which GABAergic TRN neurons they innervate. In addition, ThalamoCortical matrix neurons, such as those in the CentroMedial Thalamic (CMT) nucleus or those among matrix cells that sheath thalamic nuclei, project diffusely to the cortex. These neurons mostly innervate cortical layer I, although they also project to deeper cortical layers and the striatum [70].

The CorticoThalamic neurons are located within layers V and VI [71], also named IFL. The IFL mainly comprises pyramidal neurons (~70–80 % of all cortical neurons). Layer VI CorticoThalamic neurons send localized excitatory inputs to the thalamus, while layer V neurons send diffuse excitatory projections [72]. Layer VI CorticoThalamic neurons also send inhibitory projections to the pyramidal neurons in layer IV, which express inhibitory metabotropic glutamate receptors. The glutamatergic CorticoThalamic neurons provide intra-cortical inhibition while sending excitatory inputs to GABAergic neurons of the TRN and ThalamoCortical relay neurons, acting as a critical candidate in the synchronization of CorticoThalamic networks and the dysregulation thereof as observed in TCD and psychedelic-induced states. [73]

Box 1 The thalamic reticular nucleus (TRN): the gatekeeper of the thalamus and rhythm generator By providing powerful inhibition to ThalamoCortical relay cells and filtering the ThalamoCortical information flow, the TRN is often referred to as the "gatekeeper of the thalamic gate". TRN cells exhibiting high level of activity that can inhibit almost entirely ThalamoCortical relay neurons. In this state (the thalamic gate is closed), no information is relayed to the cortex. On the contrary, when the GABAergic neurons of the TRN themselves are inhibited, ThalamoCortical relay neurons are active, and ThalamoCortical neurotransmission is allowed (the thalamic gate is open). Due to its intrinsic pacemaking properties, the TRN has been considered the "brain rhythm generator". The vast array of highly specialized electrophysiological properties of GABAeric neurons of the TRN provides the ideal conditions to fulfill this role. Furthermore, the TRN is enriched in 5-HT2A receptors [217], explaining the reliance on the thalamus for psychedelic effects.

### Thalamocortical dysrhythmia [74]

ThalamoCortical networks exhibit two primary distinct states: synchronized oscillatory activity, mainly observed during sleep, which blocks the relay of sensory information to the cortex, and tonic activity, which allows ThalamoCortical information flow and is observed during wake and REM sleep. Due to the ability to allow or block the flow of information generated from external and internal stimuli, the thalamus is the ideal structure for the modulation of sleep and arousal [75]. The release of neurotransmitters throughout ThalamoCortical networks from the brainstem determines which state the brain is operating in and allows the transition from sleep to arousal [76].

#### The thalamus, a key target of PD-related pathology

A growing body of recent evidence indicates that the driver for the altered network activities driven by synuclein resides in the thalamus. The region modulates altered sensory filtering and generates thalamic rhythms associated with the TCD complex [77, 78].

TCD is characterized by low-threshold bursting activity that exhibits a shift from the alpha to theta rhythmicity in thalamic neurons [77, 78]. This is a typical electroencephalography (EEG) activity observed during drowsiness states [79] and a shift from resting-alpha activity which is replaced by constant cross-frequency coupling of low- (theta frequencies, 4–8 Hz) and high-frequency (gamma, 25–50 Hz) oscillations [78]. While theta oscillations are thought to underlie negative symptoms (such as depression and hearing loss), gamma frequencies have been linked to the production of positive symptoms (such as pain and tinnitus) [78].

The TCD model postulates that the thalamic bursting activity is associated with vigilance, while the thalamic slow oscillatory activity is associated with sleep phases [77, 80]. In the original TCD model [77], slow thalamic activity in PD patients was associated with long-lasting hyperpolarization generated from altered inputs projecting from the pedunculopontine nuclei or striatum to the TRN. The hyperpolarization of TRN activates slow CorticoThalamic oscillations generated from high-order thalamic nuclei [81]. These oscillations are produced by the sequential de-inactivation of T-channels and by low-threshold calcium spiking activity that ensues upon T-channels dysfunction. The spiking activity has a frequency in the theta range that produces a shift to a dysrhythmic state. The TCD model is based on scalp EEG recordings and MEG data [77]. Furthermore, the hyperpolarization of ThalamoCortical relay neurons driven by decreased excitatory inputs may ultimately shift their activity to theta fire bursts. The process generates a feed-forward self-perpetuating loop. In addition, a “theta cross-modular spread” occurs through divergent projections.

Supporting a role for TCD in the modulation of consciousness, subcortical stereo EEG recordings have indicated that state-dissociations or state-switches are associated with rhythmic, spindling activity in the theta range generated by the thalamus [45, 80, 82–84]. The electrical alterations described above should be considered in the context of the complex cognitive activities exerted by the thalamus. The thalamic role in shaping cognition has been recently conceptualized within the “cognitive thalamus” hypothesis [85]. The theory refers to the modulation of Cortico-Thalamo-Cortical interactions exerted by the high-order thalamic nuclei’s composite structure [72, 86–88], which may be crucial for specific cognitive processes and global functioning. For instance, the MDT (see Box 2) participates in memory, attention, and emotion processes. Thus, damage of this structure may cause severe functional impairment and trigger confabulations and various cognitive deficits [89, 90].

A recent meta-analysis study [91] assessing region-specific effects of acetylcholine agonists revealed that the pharmacological activation of the cholinergic system produces increased activity in the lateral frontoparietal regions, thalamus, and cuneus as well as enhanced DMN deactivation. In line with those findings and the model presented here, cholinergic inputs from the brainstem reticular formation excite ThalamoCortical relay neurons while inhibiting the GABAergic TRN neurons. The process is in stark contrast to CorticoThalamic excitatory inputs, which provide excitatory input to both ThalamoCortical neurons and the GABAergic neurons of the TRN that inhibit the former. Thus, it is conceivable that the dysregulation of cholinergic neurotransmission from the brainstem reticular formation leads to pathological disinhibition of ThalamoCortical relay neurons. The consequent allostatic load on the ThalamoCortical system may contribute to the dysregulation of ThalamoCortical pathways, thereby promoting the onset of TCD.

**Fig. 1:**
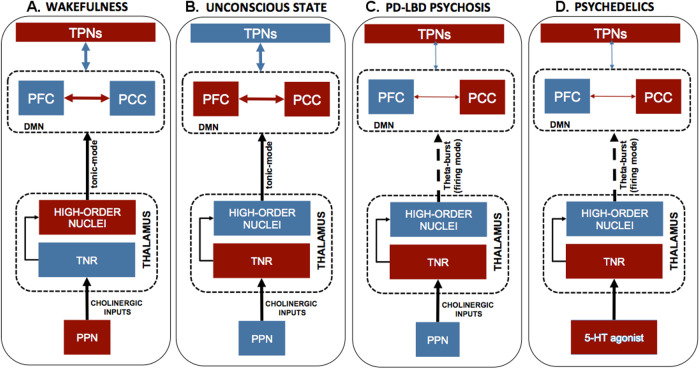
Network changes occurring upon the PD-DLB psychosis complex and psychedelics. **A** Upon physiological conditions, the cholinergic transmission from the pedunculopontine nuclei inhibits the thalamic reticular nuclei, activates the high-order thalamic nuclei, and produces high-frequency cortical oscillations (tonic mode). These activities suppress DMN functioning and increase TPN engagement. **B** Upon unconsciousness or during REM sleep, the down-regulation of cholinergic projections from the pedunculopontine nuclei to the thalamic reticular nuclei deactivates the high-order thalamic nuclei, promotes low-frequency oscillations (burst-firing mode), and decreases the thalamic control exerted on the DMN. **C**, **D** Pathological pedunculopontine nuclei changes in PD/DLB patients or psychedelic-driven 5-HT modulation disinhibits the thalamic reticular nuclei. The overactivated thalamic reticular nuclei inhibit the high-order thalamic nuclei and produce low-frequency oscillations (burst-firing mode), thereby uncoupling the DMN by decreasing the thalamic control of the network. The red and blue colors indicate down- or up-regulation of the brain systems, respectively. DLB, Dementia with Lewy Bodies, DMN Default Mode Network, MD mediodorsal nuclei, PD Parkinson’s Disease, PN posterior nuclei, PPN peduncolopontine nuclei, TPNs task-positive networks, TRN thalamic reticular nuclei.

Finally, a complementary critical process that drives the thalamocortical interactions is located in the pedunculopontine nucleus [92]. Cholinergic neurons control the Thalamo-Cortical excitability and behavioral outputs within the pedunculopontine nuclei. Pedunculopontine nuclei neurons inhibit the TRN, activate ThalamoCortical neurons, and promote high-frequency cortical oscillations associated with wakefulness [93–95]. Thus, cholinergic inputs, projecting from the pedunculopontine nuclei to the thalamus, critically participate in the transitions between wakefulness and REM sleep [94, 96, 97] (Fig. 1).

Box 2 The MedioDorsal Thalamus (MDT): processor and relay to the cortex The MDT receives inputs from the amygdala and olfactory cortex and projects to the PFC and the limbic system and, in turn, relays them to the PFC network allowing a fine-tuned control of those excitatory inputs, which is required for the spatiotemporal precise transfer of relevant ThalamoCortical information. Depending on their membrane voltage, which is at least partially determined by driver and modulatory CorticoThalamic inputs, ThalamoCortical relay neurons operate in diverse functional modes, thereby promoting network oscillations and synchronizing low- or high-frequency ThalamoCortical oscillations [67]. This capability has been attributed to the underlying Ca^2+^-dependent low-threshold spike, mediated by voltage-gated calcium channels present in TC and TRN neurons.

### ThalamoCortical imbalance: role of neuropathological changes in PD-DLB

Pathological aggregates of α-synuclein in eosinophilic Lewy bodies are the hallmark of synucleinopathies like PD and DLB [98]. α-synuclein is an intracellular protein involved in presynaptic terminal functioning [99]. In synucleinopathies, the pathological subcortical spreading of α-synuclein through intrinsic networks and projections to the cortex underlies disease propagation, a process possibly occurring in synergy with amyloid deposition [100, 101]. Such spreading occurs stereotypically along structural and functional connections connecting the brainstem to the cortex [101, 102].

A recent review has detailed the distribution of α-synuclein aggregates in the disease [103]. The thalamus and the reticular-thalamo-cortical activating system are the preferential targets for synuclein-related pathology [102, 104–106]. In contrast, first-order nuclei, like the lateral geniculate nuclei, do not show significant accumulation of Lewy bodies or Lewy neurites and are spared from structural damage [107].

In DLB patients, the atrophy of the frontal [108], premotor [109], and posterior [110–113] regions is associated with microstructural alterations of the corresponding high-order thalamic nuclei [114, 115], including the intralaminar sub-regions, the medial and lateral portions of the pulvinar [104, 116–118]. The microstructural damage of the posterior cortices has been reported to also spread to the parieto-occipital tracts [107, 119–121], the anterior thalamic radiation [115], and the inferior longitudinal fascicle [122–124]. Frontal atrophy is also found in PD patients and is associated with the development of progressive microstructural damage in the putamen [125], subcortical white matter [126], and pedunculopontine nuclei [127]. Atrophy of the latter structure is typically found in PD patients suffering from VH [128].

These pathological findings align with recent theories that envision the frontal control system as a weak point for the ensuing neurodegenerative processes due to phylogenetic reasons [129]. The construct is based on the notion that the prosencephalon - and the frontal lobes in particular - are phylogenetically newer and have an increased relative volume compared to “older” structures in subcortical systems [129, 130]. Older structures are not equipped with an array of modulatory neurotransmitters to appropriately match the demanding functions of the frontal lobes, thereby making these regions more vulnerable to *noxae* and neurodegenerative processes [129, 130].

In line with the role played by TCD in the disease, in PD patients, theta rhythms initially appear as a pseudo-periodic intrusion on resting-state background activity and later spread out to the whole cortex [78, 131–133]. EEG abnormalities with TCD-like frequencies also occur in DLB patients. They are now employed as a diagnostic biomarker of the disease (the theta activity is, for diagnostic purposes, termed pre-alpha) [42]. These electric alterations also predict incoming cognitive decline in PD patients who already exhibit signs of mild cognitive impairment [131] and appear to be driven by the dose-dependent activation of metabotropic glutamate receptor class Ia (mGlur1a) [79]. Furthermore, in DLB patients, theta activity correlates with the severity of cognitive fluctuations [131, 134]. The theta rhythms found in PD or DLB patients initially appear on frontal derivations and then migrate posteriorly [34]. Stable pre-alpha rhythms or pseudo-periodic pre-alpha intrusions have been detected in patients exhibiting hallucinations, FND-SSD, or delusions [21, 22, 132]. Intruding pre-alpha activity also occurs in the REM sleep of PD patients [135, 136].

Interestingly, aside from PD and AD, TCD is also observed in several psychiatric disorders, such as depression, obsessive-compulsive disorder, schizophrenia, and neurogenic pain [77, 137] (in the latter, increased theta frequencies are associated with high GABA levels [138]; central lateral thalamotomy has been shown to reverse this dysfunction in humans and decreases pain [139]). Of note, schizophrenic patients – who typically exhibit vivid psychotic symptoms – display reduced thalamic volumes and blood flow, along with a dysfunction of prefrontal regions. A seed-based rs-fMRI study centered on MDT FC in schizophrenic patients found significantly decreased connections with several regions, including the middle frontal cortex, the ACC, the insula, and the cerebellum [88, 140].

### ThalamoCortical imbalance: role of serotoninergic modulation

Serotonergic 5-HT2A receptors are abundantly expressed in the V layer of the cortex and project to the thalamus. To a lesser extent, the receptors are expressed in the thalamus [141, 142]. Since 5-HT2A receptors are also expressed pre-synaptically at this crucial junction point, they may represent the molecular “missing link” between the ThalamoCortical circuits and the onset of VH. In that respect, 5-HT2A receptors may promote, as in organic psychosis, a functional overload of the circuitry (i.e., TCD) and lead to disrupted integration of sensory information as a consequence of abnormal cortico-cortical interactions [66, 143–145]. For instance, the administration of serotonergic psychedelic compounds acting on these receptors has been linked to metabolic changes and alterations of functional connectivity (see paragraphs 5.2 and 5.3) [146, 147], as observed in PD-DLB psychosis (Fig. 1).

Aside from 5-HT2A and D2 receptors, 5HT1A receptors can also produce these effects through the modulation of IFL CorticoThalamic and pyramidal neurons, which provide the majority of excitatory inputs to the TRN [148, 149]. Bottom-up filtering of the system is therefore decreased, and hallucinations are unleashed, largely overlapping with what is observed in PD-DLB patients.

#### Serotonergic system modulation in synucleinopathies

Evidence for the involvement of the serotonergic system in PD-DLB psychosis onset originally arose from pharmaco-imaging studies and clinical practice, given the role of atypical antipsychotics in managing parkinsonian psychosis [150]. Atypical antipsychotics inhibit high-affinity 5-HT2A receptors and, with relatively lower affinity, D2 dopaminergic receptors [151]. Despite some disagreement on the relative weight of the serotonergic versus dopaminergic modulation of symptoms [152], 5-HT receptors are now considered the primary pharmacological target for psychosis treatment [153]. Atypical antipsychotics like pimavanserin (exhibiting a prevalent “anti-serotonergic” profile with negligible effects on dopamine receptors) are now entering the PD-related pharmacopeia [153] along with other established atypical agents (e.g., quetiapine, clozapine) already employed as first-line therapies for parkinsonian psychosis [36, 150].

More recent evidence indicates serotonergic system imbalance in PD patients (see paragraph 4.4.2). The process involves the ventral striatum and the thalamus, but also anterior cortical regions like the orbitofrontal cortex, the anterior cingulate cortex (ACC), and the insula [154]. Similar changes have also been linked to mood disorders and, interestingly, VH onset [36]. A small case-control study indicated that compared to non-hallucinating PD subjects, PD patients with VH exhibited increased expression of 5-HT2A receptors within the ventral visual pathway (along with the insula, the dorsolateral prefrontal cortex, and the medial orbitofrontal cortex). These findings confirm the close relationship between the serotonergic system, visual function, and its alterations as observed in VH onset of PD patients [155]. These findings are also well-aligned with the PD-related neurodegeneration that affects the raphe nuclei in patients at Braak stage two [102].

#### Serotonergic system modulation by psychedelics

Serotonergic psychedelics (such as LSD, psilocybin, and DMT) induce mind and consciousness alteration, such as ego dissolution, dissociation, hallucinations, delusions, distortion of perception, and distortion in time and space. For these reasons, in animal research, LSD (high doses, more than 200 mcg/kg) is considered a model of psychosis since it mimics symptoms and neurochemical changes observed in human psychosis [156, 157].

Interestingly, serotonergic psychedelics and dissociative anesthetics (such as the NMDA receptor antagonist ketamine) exert a profound action over thalamic gating and Cortico-Thalamo-Cortical neurotransmission [145, 158, 159]. LSD-driven alterations in all dimensions of the Five Dimensional Altered States of Consciousness scale (i.e., oceanic boundlessness, visionary restructuralization, and anxious ego dissolution) are associated with increased ThalamoCortical functional connectivity [160]. On the other hand, upon LSD and psilocybin intake, decreased cerebral blood flow and BOLD signals in the thalamus, the ACC, and the PCC have been reported [161, 162]. Further observations have reconciled these apparent inconsistencies. While LSD was found to bilaterally reduce the functional connectivity of associative areas (i.e., prefrontal cortex, cingulum, insula, and temporoparietal junction), the compound simultaneously enhances the connectivity of sensory, somatomotor, and thalamic networks. Thus, these findings align with previous studies where increased or decreased thalamo-cortex connectivity were observed after psychedelics [161].

The state of “ego dissolution”, observed with psychedelics is linked to an increased global thalamic connectivity [163] and brain entropy, which is deemed opposed to the “entropy suppression” state, which underlies the waking conscious experience [62].

These results are in line with the role played by the thalamus in the modulation of consciousness through the integration between the parietal cortex, striatum, and the thalamus itself [164] and via a layer-specific control of cortical activity [34, 165] Moreover, the TRN, MDT, and IFL (especially layer V pyramidal neurons), which are rich in 5-HT2A and D2 receptors, confirm psychedelic’s neurobiological effects through the activation of these receptors in the thalamic regions [162, 166].

A recent fMRI study with LSD, psilocybin, and amphetamine has confirmed that psychedelics, but not amphetamine, increase the bottom-up and decreased top-down information flow between the thalamus and some unimodal cortices [74].

Using in vivo electrophysiology in laboratory animals, we have recently investigated the complex effect of LSD on the thalamic nuclei [166]. On one hand, LSD acutely increases the activity of a population of TNR neurons, resulting in excitation of the Infralimbic medial prefrontal cortex. On the other hand, LSD acutely decreases the activity of local GABAergic circuitries of the TRN while increasing activity in the MDT, which is a higher-order thalamic nucleus involved in cognition and sensory processing. This opposing effect of LSD in the TRN, but not in the MDT, supports the data showed by Vollenweider and Preller [159], who reported dual effects in the thalamus-cortex circuits. LSD increases the connectivity of the sensory pathways while decreasing the connectivity of the cognitive-integrative pathways. The process may lead to an inhibition of the spontaneous firing of pyramidal and CorticoThalamic cell firing (in line with the decreased DMN activity observed in neuroimaging studies).

It is possible that the decreased firing activity of TRN GABAergic neurons leads to the disinhibition of ThalamoCortical relay neurons and enhances the ThalamoCortical information flow, reinforcing this loop [158].

Another dissociative drug, ketamine, modulates the activity of TRN GABAergic neurons and ThalamoCortical neurons of the MDT, thereby leading to altered thalamic filtering reminiscent of REM sleep. Ketamine increases the firing frequency band of ThalamoCortical neurons from 5–20 Hz to 15–30 Hz while generating aberrant and generalized gamma oscillations in cortical and subcortical structures, as observed in the schizophrenic brain [167]. Notably, the authors detected TCD between ThalamoCortical neurons in terms of firing rates and network frequency oscillations, thereby supporting the notion that TCD generates dissociative states under ketamine, similar to PD-DLB [168]. In human fMRI studies, ketamine increases the CorticoThalamic connectivity of the (1) somatosensory cortex, connected with ventrolateral and ventral anterior thalamic areas; and (2) the temporal cortex connected with mediodorsal, antero-ventral, and lateral thalamic structures [169].

In the cortical EEG, ketamine generates a transient, marked decrease in theta and delta oscillations, spindle density, and power. The activity is likely dependent on a cholinomimetic activity exerted within nodes of the medial pontine reticular formation, as the cholinergic drug physostigmine induces similar effects [168, 170]. On the contrary, gamma enhancement and higher frequency oscillations after ketamine intake are due to other neurotransmitter systems [168].

Together, neuropharmacological, neuroimaging, and neuropathological data corroborate the notion that PD-DLB psychosis complex and psychedelic drugs share common neurobiological pathways within the serotonergic system, whose impairment or modulation may lead to a state of TCD. However, since psychedelics like LSD at high doses decrease the DA firing activity of the Ventral Tegmental area [156], we cannot rule out the role of a depression of mesolimbic DA in the pathogenesis of psychosis-LSD induced.

We propose that the primary functional target of TCD is the derangement of the physiological balance of network activity. Specifically, we propose that disease- or drug-driven TCD critically affects the interaction and anti-correlation between TPN and DMN, thereby unleashing the activity of the latter and generating psychotic symptoms. However, a preclinical study [171] pointed out that the modulation of the PFC by the 5-HT2A receptor stimulation produces excitatory postsynaptic potentials (EPSPs) in layer V of the mPFC, thus promoting a release of glutamate in the apical dendritic region of layer V pyramidal cells, which in turn activates the AMPA receptors. This double activation of 5-HT2A and AMPA in PFC by the LSD has been recently confirmed [172]. Brain imaging studies have also indicated the involvement of the prefrontal cortex by psychedelics, which could, per se, also explain the cognitive and mind changes induced by these compounds [146, 161].

## DMN, the critical switch of consciousness

### Definition and function of DMN

**Fig. 2:**
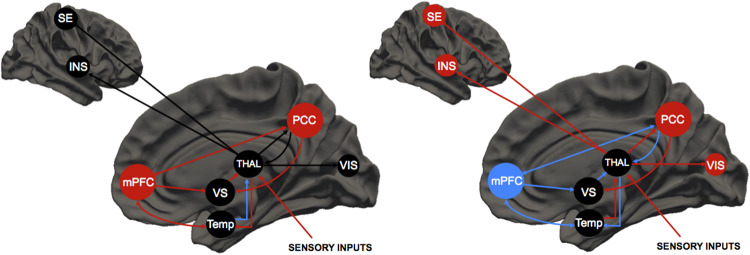
Model for psychosis upon PD-DLB or psychedelics. **Left panel:** Upon physiological conditions, the prefrontal cortex modulates the activity of the ventral striatum and temporal structures like the amygdala and parahippocampus, thereby filtering correct responses to incoming somatosensory stimuli. For instance, the amygdala shapes the integration of body perceptions and cognitive-affective information upon sensory processing. The ventral striatum modulates thalamic filtering of first-order nuclei and the physiological flux of information that, via direct connections or indirectly, via the insula, gain access to the somatosensory cortex. The thalamic high-order nuclei are relevant to shape cortico-cortical interactions, particularly connections within the DMN or between the DMN and TPN. **Right panel** Upon psychotic conditions associated with PD-DLB or triggered by psychedelics, the deregulation of the prefrontal cortex generates aberrant activity within the ventral striatum and the abovementioned temporal structures. The result is the production of out-of-control responses to incoming somatosensory stimuli and imbalanced integration of body perceptions and cognitive-affective information that occurs upon sensory processing. The dysfunctional activity of the ventral striatum decreases the thalamic filtering of first-order nuclei. It favors the production of an abnormal flux of information to the sensory cortices and triggers enhanced responses to peripheral stimuli and enhanced sensory coding of stimuli. The activation of 5-HT_2A_ receptors by psychedelics or cholinergic imbalance along the thalamo-PPN pathway promotes thalamo-cortical dysrhythmia, which increases the overactivation of the PCC. This cascade of events induces postero-frontal disconnections within the DMN, reinforces frontal dysfunction, and decouples the DMN from TPN. The red and blue colors indicate down- or up-regulation of the brain systems, respectively. DLB Dementia with Lewy Bodies, mPFC ventro-medial prefrontal cortex, PCC posterior cingulate cortex, PD Parkinson’s Disease, SE sensorimotor cortex, THAL thalamus, VS ventral striatum, Temp temporal structure, VIS visual cortex.

The uninterrupted and sophisticated prediction of the environment and events elaborated by the brain relies upon higher-order brain regions. Several observations point towards a critical role for the DMN. The DMN is a neural network encompassing a set of cortical and subcortical areas that include the PCC, the medial Prefrontal Cortex (mPFC), the angular gyri, the hippocampus, the temporoparietal junction, the medial portion of the caudate, the posterior regions of the putamen, and the thalamus [173, 174]. The DMN, which is named after its deactivation upon task execution and high levels of activity at rest, is involved in multiple functions related to self-relevant and internally directed information processing [173, 175]. DMN activity is counterbalanced by the activation of the TPN [67, 176] (Fig. 2a).

The strength of the anti-correlation of the activities of DMN and TPN is critical to preserving the so-called brain “metastability” [177, 178], which represents the dynamic and continuous neural oscillations from among different cognitive states [177].

Within the DMN, the PCC plays a crucial role in the functioning of the network. The PCC is a highly connected and metabolically active region [179] that shows transitional connectivity patterns with the TPN. The region is structurally and functionally connected with the thalamus and divided into dorsal and ventral portions [179]. The dorsal PCC has been implicated in the modulation of autonomic arousal and alertness and monitoring behaviorally relevant stimuli [179]. The overactivation of the dorsal PCC is associated with the intrusion of introspective and adaptive mental activities into task performance [180, 181]. The ventral portion of the PCC acts as the central hub of the DMN [182], shapes internally-directed thoughts, and maintains levels of functional connectivity associated with consciousness [179]. Furthermore, the ventral PCC and retrosplenial cortex activation is associated with volitional efforts to retrieve autobiographical information from memory [183] and maintain a narrow internal focus of attention [179].

### Network abnormalities in synucleinopathies

Several studies found increased connectivity within the DMN in synucleinopathies, a phenomenon particularly prominent in the PCC and its targets. In PD patients, the PCC increases its connectivity with the inferior parietal cortex [21] or primary visual system [26, 65]. Altered functional connectivity also involves the Cerebello-Thalamo-Cortical networks [184, 185], the striatum [186, 187], and the sensorimotor cortex [188]. In DLB patients, the PCC is hyperconnected with the putamen, inferior parietal lobule, cerebellum, ACC, and striatum [23, 24]. In line with this, single-photon emission computed tomography and FluoroDeoxyglucose positron emission tomography studies have revealed the presence of increased perfusion [189] or metabolism [190] in the PCC of DLB patients. This phenomenon, termed the “*Cingulate island sign”*, contrasts with the reduced perfusion observed in Alzheimer’s disease and is now a supportive diagnostic element for DLB [190, 191].

**Fig. 3:**
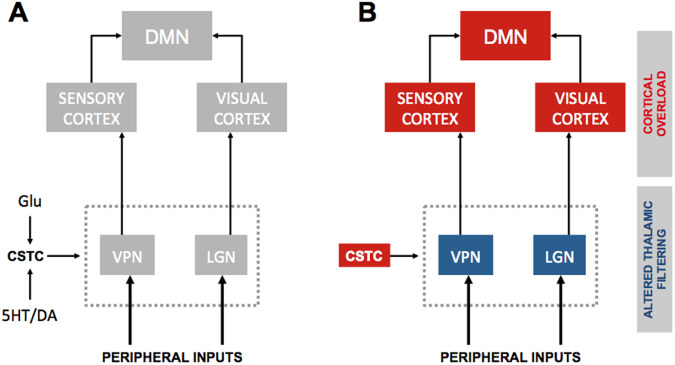
Decreased thalamic filtering in Parkinson’s disease-DLB. **A** The scheme depicts the cortico-striato-thalamo-cortical loop that physiologically controls the gating activity exerted by the first-order thalamic nuclei (ventral posterior and lateral geniculate nuclei) on external inputs. Upon physiological conditions, the cortico-striato-thalamo-cortical activity is positively modulated by glutamatergic projections that descend from the prefrontal cortex and are inhibited by the mesolimbic dopaminergic or serotoninergic projections originating from the midbrain. **B** The dysfunctional activity of the cortico-striato-thalamo-cortical loop results in decreased filtering operated by the first-order thalamic nuclei and generates a sensory overflow to the primary cortices. The mechanism works synergistically with the TCD (see also Fig. 2, panels C, D) and contributes to the increased engagement of the DMN. The red and blue colors indicate down- or up-regulation of the brain systems, respectively. CSTC cortico-striato-thalamo-cortical loop, DLB Dementia with Lewy Bodies, DMN Default Mode Network, Glu glutamate, LGN lateral geniculate nucleus, PD Parkinson’s Disease, PFC prefrontal cortex, TCD Thalamocortical Dysrhythmia, VPN ventral posterior nucleus.

Several studies investigating the functional correlates of hallucinations have shown increased connectivity at rest or upon tasks [20–24, 26, 27, 65, 192, 193] (Fig. 2b). Similar abnormalities involving DMN core regions have also been implied in the onset of FND-SSD through the activation of subcortical motor activity patterns or motor inhibition. The activation of these regions generates motor schemes that can cause a variety of symptoms ranging from “functional” paresis to catatonia [37, 194]. Specifically, a body of imaging-based evidence indicates that the hypoactivity of the ventromedial PFC and the hyperactivation of subcortical structures like the striatum, insula, and amygdala are the neuro-functional substrates to produce FND-SSD in parkinsonism [195–198]. Furthermore, prefrontal hypoactivation and hypometabolism were also observed in schizophrenia and considered the core of a unitary cortico-cerebellar-thalamic-cortical circuit [140]. From a physio-pathological standpoint, the dysregulation of the PFC can unleash the aberrant activity of the ventral striatum and amygdala, thereby leading to enhanced responses to incoming somatosensory stimuli. The increased activity of the amygdala leads to an imbalance in the integration of body perceptions and cognitive-affective information that occurs upon sensory processing [39, 199, 200]. The ventral striatum hyperactivity reduces thalamic filtering and favors the production of an abnormal flux of information that, directly or indirectly, via the insula, reaches the somatosensory cortex (Fig. 3) [201, 202]. Of note, although impaired basal ganglia circuits are known to play a pivotal role in motor symptoms of parkinsonism, their involvement in complex cognitive and psychiatric clinical pictures seems less relevant than the thalamic ones. For instance, while a minor thalamic stroke can lead to vascular dementia, with a wide range of cognitive, psychiatric, and even sleep disorders (from which the concept of “strategic stroke”), lacunar basal ganglia infarcts seldom cause such prominent and persistent cognitive/psychiatric symptoms [203–205].

In line with this model, patients suffering from FND-SSD show altered connectivity in critical hubs of the DMN and SN, like the PCC and the insula [206–209]. The increased PCC connectivity is associated with enhanced self-reference processing [207–209]. At the same time, hyperconnectivity of the salience network promotes exaggerated responses to peripheral stimuli and enhanced sensory coding of stimuli [210], thereby activating the imbalance between the DMN and TPN [128].

### Network abnormalities driven by psychedelics

Recent experimental data indicate that the thalamus plays a synergic role with the neocortex in mediating the psychedelic experience [202, 211–213]. In particular, the cortico-striatal-thalamo-cortical (CSTC) hypothesis suggests that psychedelic intake upregulated the PFC-striatum modulation of the thalamus, altering the filtering of sensory inputs to the primary cortex [145, 159, 214]. This assumption is supported by human resting-state functional Magnetic Resonance Imaging (rs-fMRI) studies showing that LSD alters the functional connectivity (FC) of the thalamus and primary sensory cortices, including sensorimotor, auditory, and visual networks [74, 144, 163]. Using dynamic causal modeling to assess the effective connectivity between the brain structures that form the CSTC loop, [74] human imaging studies in which healthy volunteers upon LSD intake have reported a bidirectional modulation of thalamic functional connectivity. Changes include 5-HT2A-dependent variations of bidirectional connectivity between the thalamus and the PCC, decreased thalamus-temporal cortex connectivity, decreased self-inhibitory connection of the temporal cortex, 5-HT2A-independent decrease of effective connectivity from the ventral striatum to the thalamus [66], and increased resting-state functional connectivity of the thalamus with regions distributed across the brain [144] (Fig. 2b). Of note, the resting-state changes were more marked in brain areas involved with VH-visual hallucinations (such as right and left lingual gyrus, right cuneus, right cerebellum, right middle occipital gyrus, and right fusiform gyrus) and auditory hallucinations (such as right and left superior temporal gyrus, right and left insula, right inferior frontal gyrus, and right precentral gyrus). Not surprisingly, LSD also affects Thalamo-Insular resting-state functional connectivity, in line with its dreamlike subjective effects.

As for psilocybin, the administration of this substance generates a state of disrupted interplay between the medial temporal lobes and the neocortex and an association between salience network disintegration and ego-loss phenomena. Interestingly, a disconnection between the parahippocampal cortex and the rest of the brain was observed after the administration of the compound to healthy volunteers, thereby suggesting that the decoupling of the medial temporal lobes from the DMN can underlie the ego dissolution state [215]. Accordingly, psilocybin was reported to decrease the frontoparietal control system at low frequencies and increase the stability of a state of global coherence [216]. Notably, psilocybin has been reported to reduce the activity of the ACC and PCC and cause significant decreases in the positive coupling between the mPFC and the PCC, thereby supporting the idea that it can acutely induce a TCD-like state [63]. A magnetoencephalographic study reported that broadband cortical desynchronization accompanies the consciousness-altering effects of psychedelics [147]. The study found that psilocybin reduces spontaneous cortical oscillatory power from 1 to 50 Hz in posterior association cortices and 8 to 100 Hz in frontal association cortices [147]. In contrast, significant decreases in oscillatory power were seen in areas of the DMN [147].

Finally, a novel circuitry has been linked to psychedelic action. Serotonergic compounds, like psilocybin or opioids acting on κ-receptors, can impair the functional connectivity of the prefrontal-claustrum-cortical loop [211]. The claustrum expresses 5-HT2A receptors [217] and regulates the sensory binding of experiences. Thus, its pharmacological modulation provides mechanistic ground for some behavioral features triggered by psychedelic substance intake [211, 218]. The activity of the claustrum, an area rich in 5-HT2A receptors [142, 217], is coupled with thalamic, parahippocampal, and striatal engagement, but further experiments are needed to support its involvement in TCD-related mechanisms [211].

The TCD-driven release of DMN activity may result in a critical alteration of the top-down modulation of sensory and cognitive processing [219–222]. We propose that the overall operational result of this unbalanced network activity is an alteration of predictive coding.

## The predictive model

From a cognitive standpoint, the PD-DLB psychosis complex can be viewed as an alteration of the computational processes involved in predictive coding [222, 223]. According to the predictive coding (or Bayesian inference) model, the brain, acting as a hypothesis generator aimed at reducing the levels of free energy, activates the perception processing along with the generation of valid cognitive outputs by employing a combinatory process [219, 221, 223, 224]. The process compares *priors* (the inner prior knowledge of likely candidates that match a given sensory input) and actual inputs to estimate a *posterior* probability. The difference between the two factors is the prediction error, which indicates the mismatch between the predicted and actual evidence [224, 225]. According to fMRI studies, prediction errors, influenced by the precision of both *priors* and sensory inputs, enhance neural responses and salience network activity. In contrast, the confirmation of predictions restrains them [223].

Perception models are common for visual, sensory, and visceromotor sensations. According to these models, somatic sensations are integrated by the activity of top-down and bottom-up control systems and the salience network [219]. These models posit that dysfunctions of predictive coding processes promote the non-physiological prevalence of priors over sensory perceptions, thereby generating false posterior probabilities [226–231]. In that respect, hallucinations and FND-SSD arise when strong (precise) generated by high-order cortices (top-down modulation) overwhelm weak (imprecise) sensory data, leading to significant prediction errors that become inappropriately relevant [232]. Therefore, percepts such as hallucinations that are not based on matching stimuli are perceived [223].

These processes find similarities with the effects triggered by psychedelics. Serotonergic psychedelics can, at high doses, interfere with the brain hierarchy and change deeply rooted but noxious beliefs (*priors*) [158, 233–239]. According to the Relaxed Beliefs Under Psychedelics (REBUS) model proposed by Carhart-Harris and colleagues, psychedelics facilitate a reduction in the top-down information flow while generating a relative overflow of the bottom-up information (high entropy brain) [233]. In line with the model, increased Lempel–Ziv complexity scores, a proxy measure of brain entropy, have been detected in studies employing magnetoencephalography and electroencephalography in subjects exposed to psychedelic substances [240, 241]. Increased entropy has also been linked to theta and delta activity [233], a common EEG signature of DLB [42].

As for delusions, the key feature of their persistency may rely upon significantly impaired prediction error signaling. This alteration, especially in the presence of cognitive impairment, leads to aberrant learning/memory consolidation processes, mostly based on prior beliefs that contrast with incoming sensory stimuli.

In other words, delusions would arise when subjects experience *repeated* prediction errors that they try to rectify not by improving the predictions but by adapting the actual inputs to them. [221] For instance, a patient could say” *I see strangers in my house every day* (due to VH). *My family tells me that nobody is there, but I see them every day, so they must be there – and my family must be lying, maybe to hurt me”*. The persistency in time is a crucial feature of delusions, compared to other symptoms of the PD-psychosis complex. This may also account for the absence of delusions upon the acute administration of psychedelic compounds. Further studies are needed to assess the possible onset of delusions upon chronic administration.

Theories on the relationship between entropy and cognitive states were postulated decades before technological advances provided experimental support for the concept. This advancement has offered the opportunity to revisit the seminal conceptualization of metapsychology.

## Towards the resolution of the mind-brain conflict

Our model provides the opportunity to analyze and explore the network-based production of psychoses. The model supports the critical role of frontal lobe dysfunctions in these processes as a reduced control activity exerted by TPN generates defective reality-checking processing. The frontal lobe importance has been reconsidered in a study suggesting that parkinsonism can be phylogenetically dependent on the progressive “frontalization” of the cognitive processes [129].

The proposed model is an α-synuclein-based model supported by converging neuroimaging, neurophysiological, and neurochemical data and is significantly different from previous conceptualizations.

The TCD-DMN decoupling model can also serve as a blueprint to explore the functional and structural correlates and effects of the psychodynamic intervention.

The central question posed by the model concerns the applicability and generalization to other psychotic conditions. Several studies investigating psychoses unrelated to neurodegenerative disorders indicate that the DMN-TPN alterations found in these conditions resemble those found in PD-DLB patients [242]. Thus, the similarities of these conditions suggest that common network dysfunctions are producing a similar psychotic phenotype despite the presence of underlying different pathological processes.

We believe that the central role played by the DMN represents a *trait-d’union* between functional, hysteria-like, disorders and the pathology-driven behavioral symptoms found in parkinsonism and altered states of consciousness induced by psychedelics, thereby providing ground for a final reunion between the fields of neurology and psychiatry as well as a move forward in the settling of mind-brain dialectics.

Finally, psychedelics’ pharmacological modulation of consciousness and the resulting changes in DMN and ThalamoCortical connections further support the notion that these processes and structures are intimately entwined [243].

## Conclusions

The proposed TCD-based model offers a largely overlapping framework to explain the similarities in symptom presentation and progression observed in synucleinopathies, altered states of consciousness found in psychiatric disorders, and psychedelics intake.

The model also sets the stage for unraveling the complex interactions between the thalamus, cortex, and other brain structures and can help generate novel insights into the nature of human consciousness.

### Supplementary information

Supplementary Materials

## Data Availability

Not applicable.
