# Supplementary material for: The central role of the Thalamus in psychosis, lessons from neurodegenerative diseases and psychedelics
Source: Transl Psychiatry. 2023 Dec 13;13:384. doi: 10.1038/s41398-023-02691-0 (PMC10719401; doi:10.1038/s41398-023-02691-0)
Supplement: Supplementary file 1 — Supplementary Materials [file 41398_2023_2691_MOESM1_ESM.docx]

**Supplementary Materials**

1. **Phenomenological and pathophysiological divergencies in psychotic symptoms triggered by psychedelics and synucleinopathies**

“Psychotic symptoms” can be multifaceted, but in this paper, we always refer to the term as the symptom triad of visual hallucinations, delusions, and FND-SSD. A main difference between psychedelic- and neurodegeneration-driven psychoses concerns the transitory and reversible effect in the first case, except for rare, persistent, psychedelic-induced disorders.

Visual hallucinations, often combined with other modalities, can be easily driven by psychedelics in healthy subjects (1-6). Since in synucleinopathies, the hallucinatory phenomena occur in brains burdened by degeneration, where specific sensory pathways are already impaired (olfactive and taste senses), this could partially explain the lower prevalence of hallucinations in these modalities, which are not completely absent, only less common and, possibly, less noticed (7). On the other hand, parkinsonian hallucinations typically become complex, well-formed, and frequently are shaped as living beings, even known people with whom the patients verbally interact, therefore showing an involvement of the auditory modality. Nonetheless, isolated auditory hallucinations can also occur (7).

Albeit regarded as a less known (and frequent) side effect, delusions have indeed been documented upon LSD, psilocybin, and ketamine intake (8-14). Psychedelic-induced delusions typically occur during the acute effects of the drug and may subside once they wear off. However, in rare cases, particularly in individuals predisposed to mental health conditions or have a history of psychiatric disorders, these compounds can trigger more persistent psychosis or exacerbate underlying psychiatric conditions. Various factors, including individual susceptibility, dosage, and setting, can influence the onset of delusions. Additionally, the use of psychedelics in controlled research settings differs from recreational or uncontrolled use, which can further impact the likelihood and severity of such experiences. Several reports of psychedelic-driven delusions have been described in the past, although large RCTs are still needed.

References:

- 1. Kometer M, Schmidt A, Jäncke L, Vollenweider FX. Activation of serotonin 2A receptors underlies the psilocybin-induced effects on α oscillations, N170 visual-evoked potentials, and visual hallucinations. J Neurosci. 2013 Jun 19;33(25):10544-51.
  2. Hirschfeld T, Schmidt TT. Dose-response relationships of psilocybin-induced subjective experiences in humans. J Psychopharmacol. 2021 Apr;35(4):384-397. doi: 10.1177/0269881121992676.
  3. Carbonaro TM, Johnson MW, Hurwitz E, Griffiths RR. Double-blind comparison of the two hallucinogens psilocybin and dextromethorphan: similarities and differences in subjective experiences. Psychopharmacology (Berl). 2018 Feb;235(2):521-534.
  4. Powers AR 3rd, Gancsos MG, Finn ES, Morgan PT, Corlett PR. Ketamine-Induced Hallucinations. Psychopathology. 2015;48(6):376-85.
  5. Hirschfeld T, Schmidt TT. Dose-response relationships of psilocybin-induced subjective experiences in humans. J Psychopharmacol. 2021 Apr;35(4):384-397. doi: 10.1177/0269881121992676.
  6. Schmid Y, Enzler F, Gasser P, Grouzmann E, Preller KH, Vollenweider FX, Brenneisen R, Müller F, Borgwardt S, Liechti ME. Acute Effects of Lysergic Acid Diethylamide in Healthy Subjects. Biol Psychiatry.
  7. Kulick CV, Montgomery KM, Nirenberg MJ. Comprehensive identification of delusions and olfactory, tactile, gustatory, and minor hallucinations in Parkinson's disease psychosis. Parkinsonism Relat Disord. 2018 Sep;54:40-45. doi: 10.1016/j.parkreldis.2018.04.008. Epub 2018 Apr 4. PMID: 29653909
  8. Carhart-Harris RL, Kaelen M, Bolstridge M, Williams TM, Williams LT, Underwood R, Feilding A, Nutt DJ. The paradoxical psychological effects of lysergic acid diethylamide (LSD). Psychol Med. 2016 May;46(7):1379-90.
  9. Corlett PR, Honey GD, Fletcher PC. Prediction error, ketamine, and psychosis: An updated model. J Psychopharmacol. 2016 Nov;30(11):1145-1155.
  10. Stone J, Kotoula V, Dietrich C, De Simoni S, Krystal JH, Mehta MA. Perceptual distortions and delusional thinking following ketamine administration are related to increased pharmacological MRI signal changes in the parietal lobe. J Psychopharmacol. 2015 Sep;29(9):1025-8.
  11. Griffiths RR, Johnson MW, Richards WA, Richards BD, McCann U, Jesse R. Psilocybin occasioned mystical-type experiences: immediate and persisting dose-related effects. Psychopharmacology (Berl). 2011 Dec;218(4):649-65.
  12. Lake CR, Stirba AL, Kinneman RE Jr, Carlson B, Holloway HC. Mania associated with LSD ingestion. Am J Psychiatry. 1981 Nov;138(11):1508-9.
  13. Bowers MB Jr. Acute psychosis induced by psychotomimetic drug abuse. I. Clinical findings. Arch Gen Psychiatry. 1972 Oct;27(4):437-40.
  14. Powers AR 3rd, Gancsos MG, Finn ES, Morgan PT, Corlett PR. Ketamine-Induced Hallucinations. Psychopathology. 2015;48(6):376-85.

1. **The thalamic pacemaker**

The resonant columns include thalamic neurons from the specific thalamic nuclei and glutamatergic cortical neurons (mostly within the VI cortical layer) (1, 2). However, while the thalamus specifically works as a pacemaker for cortical functioning, the contrary is not true (3). Thus, on occasion, the cortical "pace" can prevail on the thalamic rhythm, but in the absence of structural or physiological abnormalities (e.g., scar tissues, developmental abnormalities, channelopathies), the thalamic pace prevails (4). However, a condition that stands between the two models is represented by sensory deafferentation (e.g., Chales-Bonnet syndrome), where the low-order cortical regions are initially hypoactivated due to input deficits (and not due to a slow thalamic pace) and subsequently show “denervation hypersensitivity” (5). This leads to an overreliance on higher-level cortices to predict the content of vision, mostly leading to misperceptions and hallucinatory phenomena (6).

1. R Llinás, U Ribary, D Jeanmonod, R Cancro, E Kronberg, J Schulman, M Zonenshayn, M Magnin, A Morel, M Siegmund, Thalamocortical dysrhythmia I.: Functional and imaging aspects, Thalamus & Related Systems, Volume 1, Issue 3, 2001, Pages 237-244, 10.1016/S1472-9288(01)00023-1
2. Llinás, R., Ribary, U., Jeanmonod, D., Cancro, R., Kronberg, E., Schulman, J., ... & Siegmund, M. (2001). Thalamocortical dysrhythmia I.-Functional and imaging aspects. Thalamus & Related Systems, 3(1), 237-244.
3. Halassa MM, Acsády L. Thalamic Inhibition: Diverse Sources, Diverse Scales. Trends Neurosci. 2016;39(10):680-693. doi:10.1016/j.tins.2016.08.001
4. Roux F, Wibral M, Singer W, Aru J, Uhlhaas PJ. The phase of thalamic alpha activity modulates cortical gamma-band activity: evidence from resting-state MEG recordings. J Neurosci. 2013;33(45):17827-17835. doi:10.1523/JNEUROSCI.5778-12.2013
5. Burke W. The neural basis of Charles Bonnet hallucinations: a hypothesis. J Neurol Neurosurg Psychiatry. 2002;73(5):535-541. doi:10.1136/jnnp.73.5.535
6. Sterzer P, Adams RA, Fletcher P, et al. The Predictive Coding Account of Psychosis. Biol Psychiatry. 2018;84(9):634-643. doi:10.1016/j.biopsych.2018.05.015
